# Biochar regulates the functions of keystone taxa to reduce *p*-coumaric acid accumulation in soil

**DOI:** 10.3389/fmicb.2024.1458185

**Published:** 2024-09-11

**Authors:** Xuanquan Zhu, Meng Jia, Dingchun Zi, Peng Zhou, Yu Du, Na Wang, Huijuan Dai, Ge Wang, Yuxiang Bai

**Affiliations:** ^1^College of Tobacco Science, Yunnan Agricultural University, Kunming, China; ^2^China Tobacco Hebei Industrial Co., Ltd., Shijiazhuang, China

**Keywords:** biochar, *p*-coumaric acid, adsorption characteristics, keystone taxa, microbial function

## Abstract

**Introduction:**

Applying biochar (BC) to reduce toxic substance accumulation in soil, either through direct adsorption or modulation of the microbial community, has received considerable attention. However, a knowledge gap exists regarding how BC regulates microbial community structure and functions to mitigate toxic substance accumulation.

**Methods:**

We previously identified p-coumaric acid (*p*-CA) as a representative autotoxin in tobacco rhizosphere soil. On this basis, this study simulated a soil environment with *p*-CA accumulation to investigate the impacts of BC on *p*-CA, soil physicochemical properties, and microbial community structure and function.

**Results:**

The results showed that *p*-CA could be directly adsorbed onto BC, which followed the pseudo-second-order kinetic model (*R*^2^ = 0.996). A pot experiment revealed that BC significantly reduced soil *p*-CA, altered soil microbial composition, and enhanced bacterial community diversity. A weighted correlation network analysis showed a close association between taxon 1 in the microbial network and *p*-CA, suggesting a pivotal role for this taxon in reducing *p*-CA, with Devosia and Nocardioides identified as potential key contributors to this process. The prediction of possible keystone taxa functions showed that BC increased the relative abundances of aromatic compound degraders. Mantel tests indicated that soil organic matter exerted the greatest influence on keystone taxa functions and hub genera.

**Discussion:**

These findings suggest that BC may either directly chemisorb *p*-CA or indirectly facilitate *p*-CA degradation by regulating the functioning of keystone taxa. The results of this study provide a novel perspective for further investigation of the mechanisms through which BC reduces the accumulation of toxic substances in soil.

## 1 Introduction

The accumulation of *p*-coumaric acid (*p*-CA), which is naturally produced by plants, has been recognized as a significant contributor to continuous cropping obstacles for crops such as strawberry (Chen et al., 2020) and alfalfa (Wang R. T. et al., 2022). We noted this phenomenon in fields with continuous tobacco plantation, and autotoxicity evaluation revealed *p*-CA as a critical factor in continuous cropping obstacles (Bai et al., 2019). The alleviation of *p*-CA accumulation is of particular importance in the context of continuous tobacco cropping in China (Ku et al., 2022; Wang R. Q. et al., 2022; Lin et al., 2023; Zhang C. et al., 2023). Current efforts to reduce *p*-CA and similar toxic substances rely primarily on chemical or biological methods (Lubbers et al., 2021; Wang Y. et al., 2021; Xie et al., 2022). However, these approaches face limited acceptance due to high cost and long treatment duration. The accumulation of *p*-CA urgently needs to be alleviated by developing simple and cost-effective techniques.

Biochar (BC), a carbon-rich substance produced by the pyrolysis of biomass at high temperatures (300°C–700°C) and under conditions of limited oxygen (Li et al., 2020; Qin et al., 2022), has been widely researched in recent years for managing contaminated soil, enhancing soil quality, and alleviating continuous cropping obstacles (Haider et al., 2022; Liu et al., 2022; Tang et al., 2022; Zhang M. et al., 2023). Studies have demonstrated that BC can decrease the amount of toxic substances in soil, either directly or indirectly. The porous structure of BC can adsorb toxic substances through electrostatic actions and intermolecular forces to prevent direct harm to crops (Lu et al., 2020; Li et al., 2021; Zheng et al., 2022). Additionally, the unique physical properties of BC create a favorable habitat for soil microorganisms (Sarfraz et al., 2020), leading to substantial alterations in the structure and function of microbial communities. This can enhance the biodegradation capabilities of microbial communities, transforming toxic substances into less toxic or more mobile forms (Rashid et al., 2022). Unfortunately, the detailed mechanisms and effects of the influence of BC on soil microbial community structures and functions remain inadequately understood.

The structure and function of microbial communities play crucial roles in maintaining soil health, with keystone taxa serving as the core components (Muhammad et al., 2021; Yang et al., 2021). Taxa are highly interconnected groups that significantly influence microbial community structure and function through collective benefits or synergistic interactions (Chen et al., 2019). Studies have shown that the abundance of keystone taxa is closely related to the overall stability of the community and, to some extent, reflects the nutrient use efficiency of the soil microbial community (Li et al., 2023). Moreover, keystone taxa are essential in the degradation of *p*-CA. Research indicates that *p*-CA in the soil can induce microbial taxa that use *p*-CA as the sole carbon source to accumulate in the rhizosphere, ultimately reducing the *p*-CA content (Blum et al., 2000; Zhou et al., 2018). Specific microbial groups, such as *Sphingobacterium* sp., can secrete extracellular enzymes (lignin peroxidase or manganese peroxidase) that degrade *p*-CA into smaller acids (e.g., butyric, propionic, oxalic, and acrylic acids) and alcohols (e.g., ethanol), thereby reducing the phytotoxicity of *p*-CA to crops (Wang et al., 2018). Studies have also shown that biochar (BC) can alter the composition and function of soil keystone taxa, enhancing their competitiveness within the microbial community, improving nutrient use efficiency, and optimizing the soil microbial structure (Wang X. Y. et al., 2021; Yan et al., 2021). However, research on the microecological effects of BC in soils containing *p*-CA, particularly regarding changes in keystone taxa and their correlation with *p*-CA degradation, remains limited.

To address the abovementioned knowledge gaps, in the present study, soil samples were prepared with *p*-CA and BC based on our previous investigations. The objective of this study is to clarify the effects of BC on *p*-CA absorption, soil physicochemical properties, and microbial communities, seeking answers to the following three questions: (1) the mechanisms of *p*-CA adsorption by BC; (2) the effects of BC on *p*-CA content, soil physicochemical properties, and keystone taxa; and (3) the potential paths and biological mechanisms through which BC reduces *p*-CA. Through this study, we can gain a better understanding of the ecological effects of BC and find new ways to investigate the mechanisms of BC in reducing toxic substances in soil.

## 2 Materials and methods

### 2.1 Experimental material

Soil samples were collected from abandoned land in the training base of Yunnan Agricultural University (25°8′N, 102°45′E). The soil was typical of the red soil found in Yunnan. Supplementary Table S1 displays the basic physiochemical properties of soil. The *p*-CA standard (99% purity) was purchased from Aladdin (Shanghai, China). The stems of tobacco variety K326 (*Nicotiana tabacum* L., McNair30 × NC95) grown with conventional cultivation methods were collected from Jiuxi Town, Yuxi City, Yunnan Province, China (24°18′N, 102°38′E). Following natural air drying, the tobacco stems were crushed, filtered through a 0.355-mm sieve, and pyrolyzed at 600°C for 6 h to yield tobacco stem BC. The physiochemical properties and microstructure of BC are shown in Supplementary Table S1 and Supplementary Figure S1.

### 2.2 Experimental design

#### 2.2.1 Adsorption of *p*-CA onto BC

Adsorption kinetics: BC suspensions were prepared by adding 5 g BC to 50 mL of 150 mg/L *p*-CA standard solution. The suspension was shaken at 180 rpm away from light on a rotating shaker at 25°C. Samples (5 mL) were collected at 15, 30, 45, 60, 75, 90, 105, 120, and 135 min. After centrifuging the samples at 1,570×*g* for 15 min, the supernatant was passed through a 0.25-μm diameter membrane. The *p*-CA level was measured using an Agilent 1,200 high-performance liquid chromatography (HPLC) system (Agilent, Santa Clara, CA, USA). The amount of *p*-CA adsorbed by BC (Qt) was calculated using Equation 1. The pseudo-second-order kinetic model (PKE) (Equation 2), the Elovich model (EM) (Equation 3), and the intra-particle diffusion model (IDM) (Equation 4) were utilized to fit the experimental data.

Adsorption isotherms: BC samples weighing 5 g were added to 50 mL *p*-CA standard solutions with concentrations of 20, 40, 60, 120, 240, and 360 mg/L. The mixtures were incubated at 25°C and 180 rpm for 12 h in the dark. The detection method for *p*-CA was similar to that used for the adsorption kinetics experiment. The experimental data were fitted using the Langmuir model (Equation 5) and the Freundlich model (Equation 6).

#### 2.2.2 Soil incubation experiment

A pot experiment was conducted in a growth chamber without BC (NB) and with 20 g/kg BC (AB). Each treatment consisted of nine replicates (three pots per replicate, with 250 g soil in each pot). The *p*-CA was added at a rate of 15 mg/kg dry soil and allowed to equilibrate for 1 week. BC was then added and thoroughly mixed with the soil. The pots were incubated in an artificial climate chamber under specific temperature and humidity conditions (Supplementary Table S2). At 30 d after BC addition, 0–2 cm of topsoil was removed, and nine samples were collected from the pot. Two samples were collected from each replicate. The soil physiochemical properties were determined after air-drying one sample, while another was stored at −80°C to detect *p*-CA residue and microorganisms in the soil.

### 2.3 Measurement of *p*-CA

The *p*-CA contents were quantified using an Agilent 1200 HPLC system (Agilent) with a SunFire™ C_18_ column (4.6 mm × 250 mm, 5 μm; Waters, Milford, MA, USA). The mobile phase consisted of methanol (A) and 1% acetic acid aqueous solution (B), and the gradient elution program and setup details are shown in Supplementary Table S3. Pure *p*-CA was used as the standard sample, with an injection volume of 10 μL. To ensure the stability and repeatability of the analysis, samples were injected at 10-min intervals to mitigate potential interference caused by impurities with extended retention times. To prepare the *p*-CA standard solution, 0.0100 g of *p*-CA was dissolved in a small amount of methanol and diluted to 1,000 mL with ultrapure water, resulting in a 10 μg/mL *p*-CA stock solution. Subsequently, the stock solution underwent dilution by factors of 5, 10, 20, 40, and 100 to generate the standard working solutions.

### 2.4 Kinetics of *p*-CA adsorption

The adsorption of *p*-CA onto BC was characterized using adsorption kinetic models and adsorption isotherm models, including PKE (Equation 2), EM (Equation 3), IDM (Equation 4), Langmuir (Equation 5), and Freundlich (Equation 6) (Tomczyk et al., 2020; Zand and Abyaneh, 2020).

The quantity (*Qt*) of *p*-CA adsorbed onto BC was calculated as follows:


(1) $$Qt=\frac{\left(C0-Ct\right)\times V}{M}\text{,}$$

where *Qt* is the quantity of adsorbed *p*-CA, mg/g; *C*_0_ is the initial concentration of *p*-CA, mg/L; *Ct* is the concentration of *p*-CA at time *t*, mg/L; *V* is the volume of *p*-CA solution, L; and *M* is the mass of BC, g.

The experimental data were fitted using the PKE (Equation 2), EM (Equation 3), and IDM (Equation 4) models:


(2) $$\frac{t}{Qt}=\frac{1}{k}Qe^{2}+\frac{1}{Qe}t\text{,}$$

(3) Qt=a+blnt,

(4) $$Qt=k_{p}t^{\frac{1}{2}}+c\text{,}$$

where *Qe* is the equilibrium adsorption capacity of *p*-CA, mg/g; *k* is the rate constant of PKE, g/(mg·min); *k_p_* is the rate constant of IDM, mg/(kg·min^1/2^); and *a*, *b*, and *c* are constants.

The Langmuir (Equation 5) and Freundlich (Equation 6) models were used to fit the adsorption isotherms:


(5) $$\frac{Ce}{Qe}=\frac{1}{\mathrm{Qmax}}Ce+\frac{1}{\mathrm{Qmax}K_{L}}\text{,}$$

where *Ce* is the equilibrium concentration, mg/L; *Qe* is the equilibrium adsorption capacity, mg/g; *Qmax* is the maximum adsorption capacity, mg/g; and *K_L_* is the Langmuir constant. The Freundlich model is calculated as follows:


(6) $$Qe=K_{F}Ce^{\frac{1}{n}}\text{,}$$

where *Ce* is the equilibrium concentration, mg/L; *Qe* is the equilibrium adsorption capacity, mg/g; and *K_F_* and *n* are the Freundlich constants.

### 2.5 Determination of soil physiochemical properties

The soil pH was measured using the potentiometric method (soil: water ratio of 1:2.5). The potassium dichromate oxidation method was employed to determine the soil organic matter (SOM) content. The molybdenum blue colorimetric method (NY/T 88-1988) was used to assess the total phosphorus (TP) content. Total nitrogen (TN) levels were quantified using the semi-micro Kjeldahl method (NY/T 53-1987). Total potassium (TK) levels were determined by fusion bonding with NaOH and subsequent measurement using a flame photometer (NY/T 87-1988), referencing the Agricultural Standards Publishing Research Center guidelines from 1987 to 2014. Available phosphorus (AP) was determined using the molybdenum-antimony resistance colorimetric method (NY/T 1121.7-2006). Alkali-hydrolytic nitrogen (AN) was measured using the alkali-diffusion method (LY/T 1229-1999) that was aligned with the DB51/T 1875-2014 standard. Finally, the soil’s available potassium (AK) content was assessed using an ammonium acetate extraction method followed by flame photometry (NY/T 889-2004).

### 2.6 Soil DNA extraction, Illumina MiSeq sequencing, and data deposition

The HiPure Soil DNA kit (Magen, Guangzhou, China) was used to extract the soil microbial DNA. The concentration and purity of DNA were determined using 1% agarose gel electrophoresis. PCR (Eastwin, Beijing, China) was used to amplify the target region of the 16S rRNA gene (16S V3–V4). The PCR reaction cycle consisted of initial denaturation at 95°C for 5 min; followed by 30 cycles of 95°C for 1 min, annealing at 60°C for 1 min, and extension at 72°C for 1 min; with a final extension at 72°C for 5 min. The reaction system consisted of 10 μL of the reaction buffer, 10 μL of the high GC enhancer, 1.5 μL of dNTPs (2.5 mM), 1.5 μL of forward and reverse primers (10 μM, 341F: CCTACGGGNGGCWGCAG and 806R: GGACTACHVGGGTATCTAAT), 0.2 μL of high-fidelity DNA polymerase, and approximately 50 ng of template DNA. Reagents that were related to PCR were obtained from New England Biolabs, MA, USA. The quality of the amplified products was assessed using 2% agarose gel, purified using AMPure XP Beads (Beckman, CA, USA), and quantified using Qubit 3.0. Sequencing libraries were constructed using the Illumina DNA Prep Kit (Illumina, CA, USA). The ABI StepOnePlus Real-Time PCR System (Life Technologies, Foster City, USA) was used to test the library quality. Qualified libraries were sequenced by Novaseq 6,000 PE250 using the pooling mode. The raw data were uploaded to the China National Center for Bioinformation (CNCB)^1^ (Chen et al., 2021; Xue et al., 2022) database (GSA: CRA013351). In the raw sequencing data, a large amount of low-quality or biologically insignificant data (such as chimeras) may have been generated due to sequencing errors. To ensure the statistical reliability and biological validity of the subsequent analyses, we first used FASTP software (v0.23.4) to remove low-quality reads. The paired-end reads were then assembled and merged into tags, and the tags were filtered to obtain the clean tags. Next, the UPARSE algorithm in USEARCH software (v10) was employed to cluster the clean tags, removing chimeric tags detected during the clustering process. The resulting data are referred to as Effective tags. Finally, the OTU abundance was calculated based on the Effective tags, resulting in 2,352 OTUs for the AB treatment and 2,033 OTUs for the NB treatment.

### 2.7 Statistical analysis

Experimental data were stored in spreadsheets in Microsoft Excel 2023. Statistical analysis was performed using the R (version 4.2.1). The Levene and Shapiro–Wilk tests were used to test the data for homogeneity of variances and normality. The difference between NB and AB was assessed using a *t*-test. (*α* = 0.05). The Chao1, Shannon, Simpson, and Ace indices were calculated from microbial sequence data using QIIME software (version 1.9.1). Differences in *α* diversity indices between groups were assessed using the R language. Microbial keystone taxa were identified using the Weighted Gene Co-expression Network Analysis (WGCNA) package (version 1.72-1). Correlation plots were generated using the ggplot2 package (version 3.4.2). The Mantel test was performed using the microeco package (version 0.20.0). The Functional Annotation of Prokaryotic Taxa (FAPROTAX, version 1.2.1) software was used to extrapolate bacterial community functions. Structural equations (SEM) were constructed using the Partial Least Squares Path Modeling (PLS-PM) package (version 0.5.0). Path coefficients were estimated using 1,000 bootstraps. To ensure the validity of the SEM results, only latent variables with loading values greater than 0.8 were retained. Correlation networks were visualized using Gephi (version 0.10).

## 3 Results

### 3.1 *p*-CA adsorption by BC

**Figure 1:**
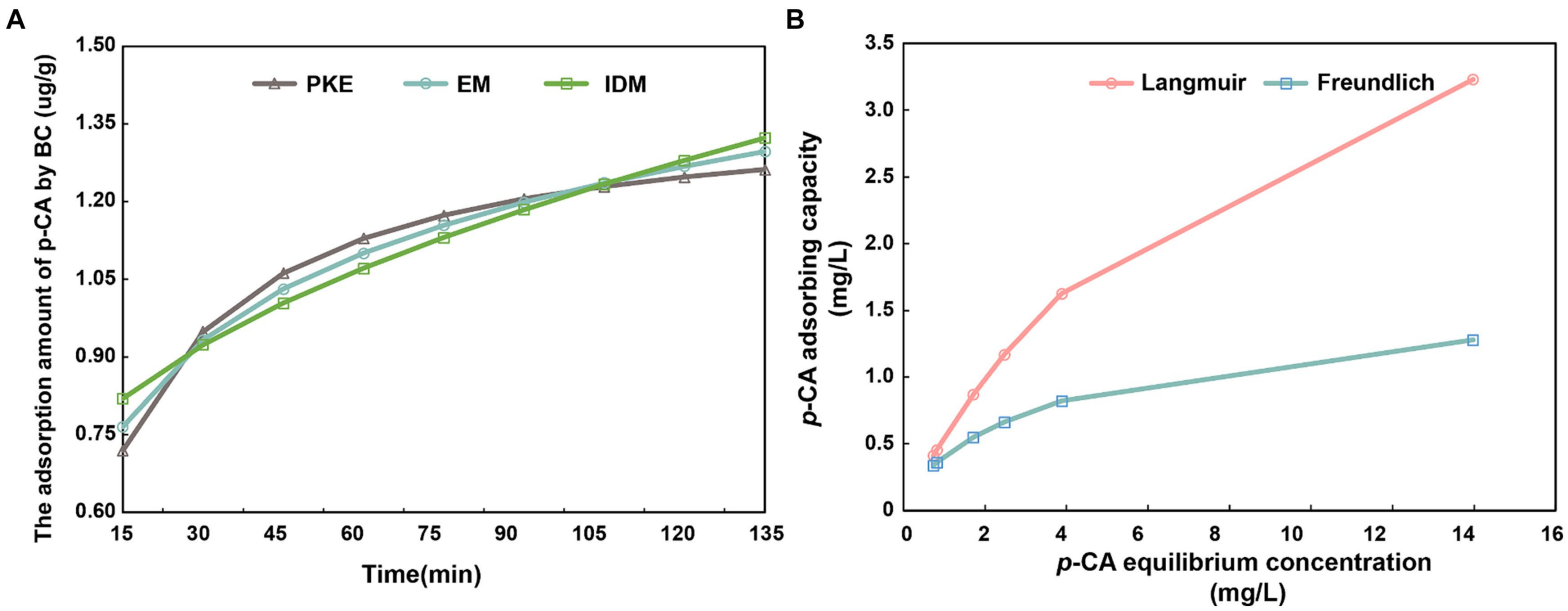
Direct adsorption of *p*-coumaric acid by biochar. **(A)** shows the regression analysis of biochar adsorption kinetics for *p*-CA using the pseudo-second-order kinetic model (PKE), Elovich model (EM), and intra-particle diffusion model (IDM). **(B)** illustrates the isothermal adsorption process of *p*-CA onto biochar, fitted using the Langmuir and Freundlich equations.

The adsorption of *p*-CA by BC was characterized using adsorption kinetic equations (Figure 1). The adsorption capacity increased initially and then plateaued after 105 min (Figure 1A). The coefficients of determination (*R*^2^) of PKE, EM, and IDM were 0.996, 0.990, and 0.935, respectively (Supplementary Table S4). Adsorption isotherm models were applied to determine the adsorption parameters, including the maximum adsorption capacity and affinity (Figure 1B). The fitted equations revealed a linear increase of adsorption at low *p*-CA concentrations, which gradually slowed at high concentrations. According to the Langmuir model, the maximum adsorption capacity of *p*-CA onto BC was 5.205 mg/g (Supplementary Table S4).

### 3.2 Soil physiochemical properties

**Figure 2:**
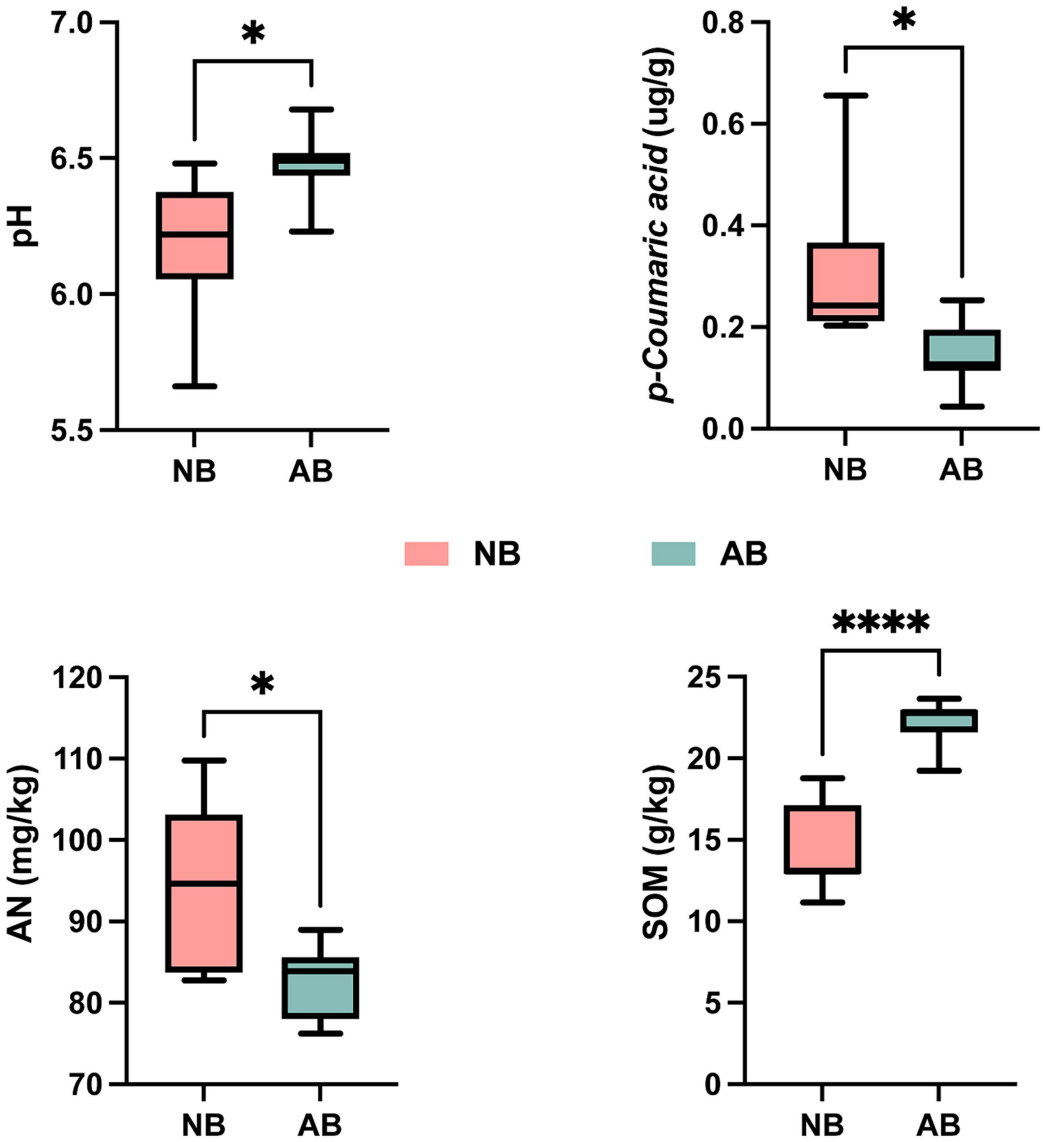
Changes in soil physicochemical properties. NB, treatment without biochar; AB, treatment with biochar; *p*-coumaric acid, *p*-coumaric acid content in soil; AN, available nitrogen in soil; SOM, soil organic matter; * denotes significant differences according to the *t*-test (*p* < 0.05), and **** denotes highly significant differences according to the *t*-test (*p* < 0.0001). The meaning of these symbols is the same for the following figures.

Compared to NB, the application of BC significantly increased soil pH and SOM by 4.52 and 55.05%, respectively (Figure 2). Conversely, *p*-CA and AN decreased significantly by 54.84 and 12.77%, respectively. Application of BC had no significant effects on TN, TP, TK, AP, or AK (Supplementary Table S5).

### 3.3 Soil microbial diversity and composition

**Figure 3:**
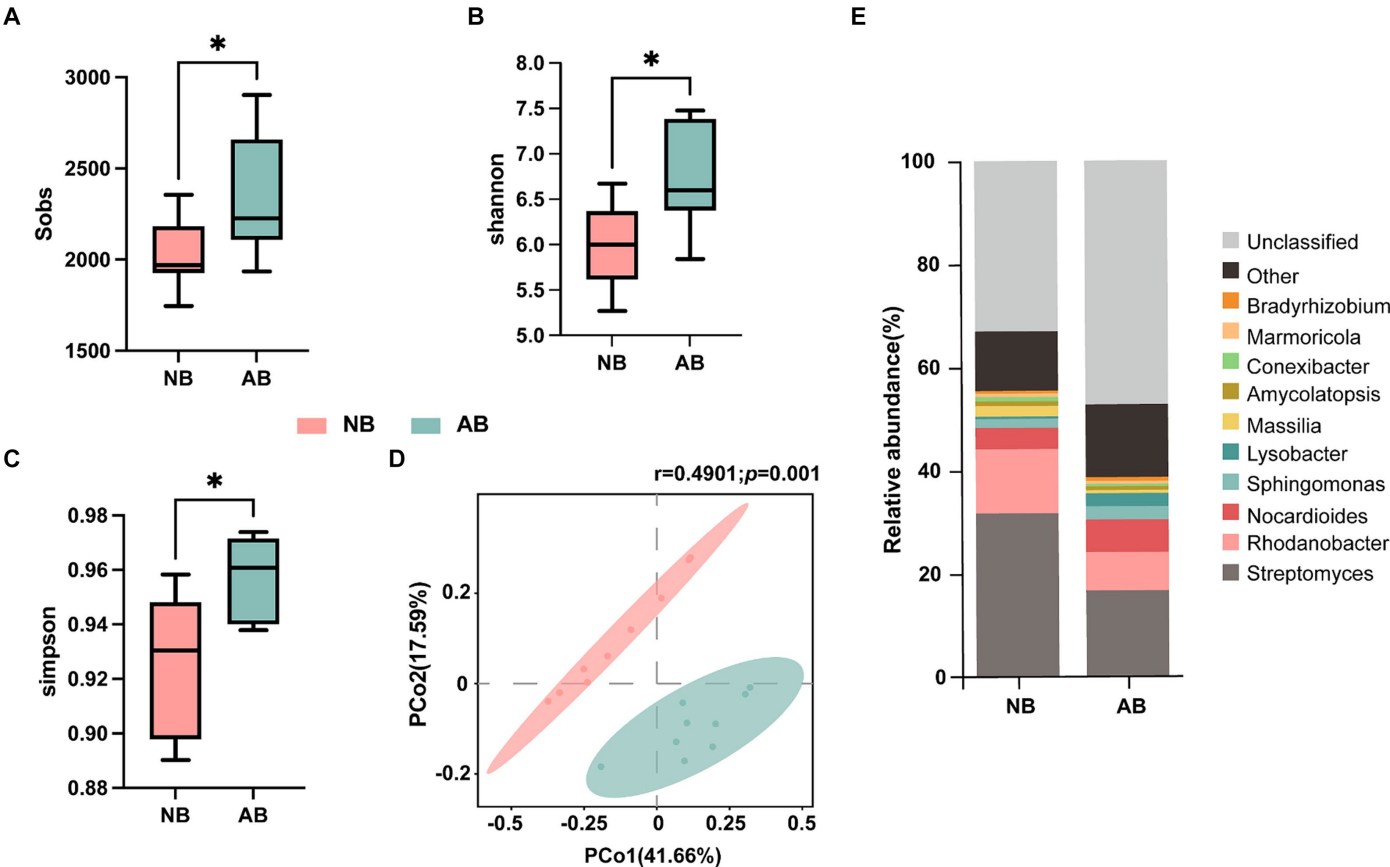
Effects of BC on soil microbial diversity. NB, treatment without biochar; AB, treatment with biochar. Panels **(A)**, **(B)**, and **(C)** illustrate the *α* diversity indices. Panel **(D)** shows the *β* diversity analysis and Adonis test; the *r* value ranging from −1 to 1 indicates within- and between-group divergence, with an *r* value close to 1 indicating a larger difference between the groups compared to the difference within the groups; and the *p* value indicates the significance of difference. Panel **(E)** shows the stacked plot of the relative abundances of the top 10 genera in the NB and AB treatments.

Variations in soil bacterial community diversity and composition were analyzed using Illumina MiSeq 16S rRNA amplicon sequencing (Figure 3). The application of BC led to significant increases in the bacterial Sobs, the Shannon index, and the Simpson index by 15.66, 12.30, and 3.47%, respectively (Figures 3A–C). Principal coordinate analysis (PCoA) based on the Bray–Curtis distance revealed significant differences in bacterial community composition following BC application (*r* = 0.4901, *p* = 0.001, Figure 3D).

In NB, *Streptomyces* (31.68%), *Rhodanobacter* (12.47%), *Nocardioides* (4.11%), and *Massilia* (2.05%) collectively constituted 50.31% of the bacterial community. In AB, the top 10 genera accounted for 38.66% of the bacterial community (Figure 3E). In addition, BC application reduced the relative abundances of *Streptomyces*, *Rhodanobacter*, and *Massilia*, while it increased the abundances of *Nocarioides* and *Lysobacter*.

### 3.4 Identification of keystone taxa and differences in their functions

**Figure 4:**
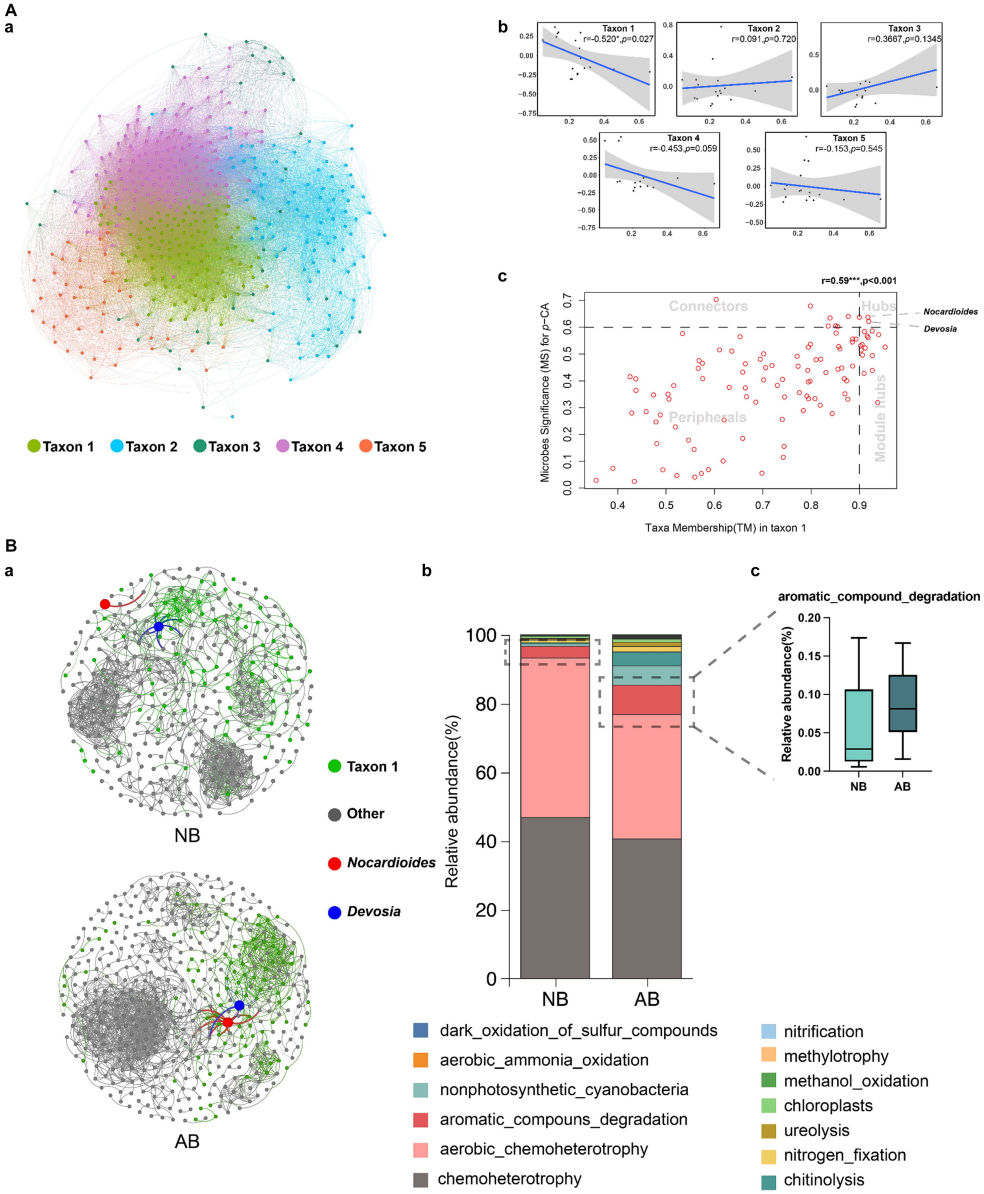
Identification of keystone taxa and differences in their functions. NB, treatment without biochar; AB, treatment with biochar; WGCNA, weighted gene co-expression network analysis; OTU, operational taxonomic unit. Panel **(A)** shows the results of WGCNA. Microbial sequencing data were filtered, and OTUs with zero tag counts were removed. Scale preprocessing was performed for data standardization. A hierarchical clustering tree was constructed without the outlier sample AB3. The soft threshold (power) was determined to be 6 based on the scale-free topology fit index >0.9 and mean connectivity >10. The network and taxa were established using a mergeCutHeight of 0.25. Panel **(A)(a)** shows the correlation network constructed using the weights calculated using WGCNA. Panel **(A)(b)** shows the correlations between taxa and *p*-*CA.* Panel **(A)(c)** shows the relationships between genera in taxon 1 and *p*-*CA.* Hubs are genera showing high correlations with other genera in the taxa and with *p*-*CA.* Taxon hubs are genera showing high correlations with other genera in the group. Connectors are genera showing high correlations with *p*-*CA.* Peripherals are peripheral genera showing low correlations with both *p*-CA and other genera in the group. Panel **(B)** shows the effects of NB and AB on keystone taxa (taxon 1). Panel **(B)(a)** shows the correlation network of taxa 1 and other groups. Spearman’s pairwise correlations with *p* > 0.9 were selected for network construction. Panel **(B)(b)** shows the different functions of taxon 1 in NB and AB. Panel **(B)(c)** shows the relative abundances of aromatic compound degradation bacteria in NB and AB.

A co-expression network was established using WGCNA. The network included five taxa (Figure 4A-a). The number and abundance of genera are presented in Supplementary Table S6 and Supplementary Figure S2, respectively. The correlation of the taxon eigenvalues and *p*-CA revealed a close relationship between taxon 1 and *p*-CA (*r* = −0.520, *p* = 0.027) (Figure 4A-b). Therefore, taxon 1 was identified as the keystone taxon for reducing *p*-CA.

The hub genera related to *p*-CA were identified by establishing the correlation between the microbe significance (MS) for *p*-CA and taxa membership (TM) in taxon 1 (Figure 4A-c). We quantified associations of individual microbes with trait of environmental factors by defining MS as the absolute value of the correlation between the microbes and the environmental factors (Langfelder and Horvath, 2008). For each taxon, we defined a quantitative measure of TM as the correlation of the taxon eigenvalue and the species abundance. The threshold values were MS = 0.6 and TM = 0.9. The analysis revealed considerable differences in the number of genera in the four regions of hubs, taxon hubs, connectors, and peripherals (Figure 4A-c). *Devosia* and *Nocardioides* demonstrated the highest correlations with other genera in taxon 1 and with *p*-CA. Therefore, *Devosia* and *Nocardioides* were selected as the hub genera.

Constructing the correlation networks of the two treatments enabled the examination of the co-occurrence patterns of the keystone taxon with and without BC. The results showed that BC application increased the number of network nodes and edges and, thus, the network density (Figure 4B-a; Supplementary Table S7). Moreover, BC altered the distribution of keystone taxa (Figure 4B-a), significantly enhancing hub genera’s relative abundances (Supplementary Figure S3). Using the FAPROTAX database, the functions of keystone taxa (taxon 1) for the two treatments were predicted (Figure 4B-b). The analysis indicated that BC application reduced the abundance of soil chemoheterotrophs and aerobic chemoheterotrophs in the keystone taxon but increased the abundance of aromatic compound degradation (ACD) bacteria in the keystone taxon (Figure 4B-c).

### 3.5 Correlations between environmental factors and microorganisms

**Figure 5:**
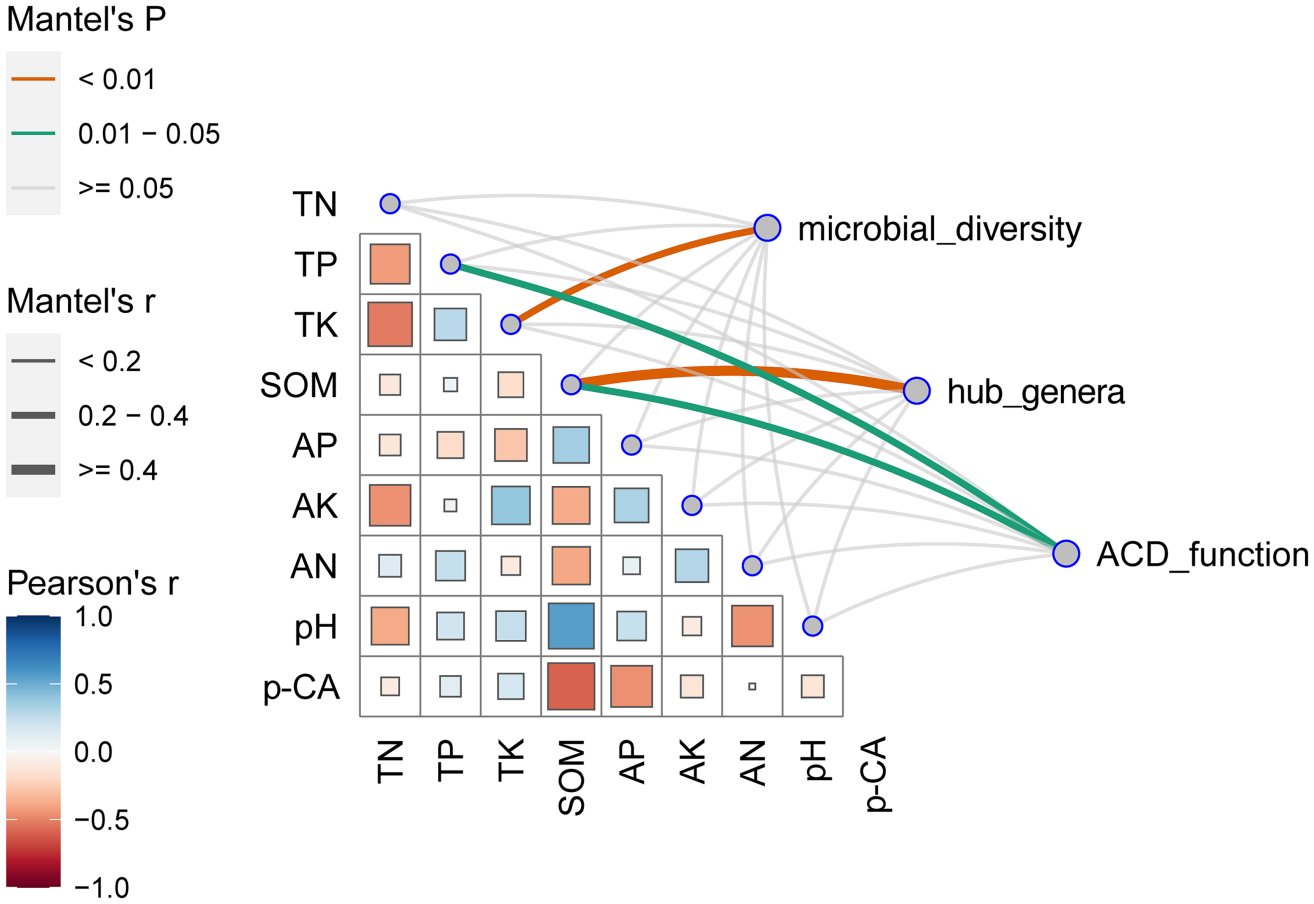
Correlations between environmental factors and microorganisms. The bottom left section shows pairwise comparisons of environmental factors, with the color gradient representing the Spearman’s correlation coefficient. The top right section displays the correlations of the microbial α-diversity (Sobs, Shannon, Simpson, Chao, ACE, Goods_coverage, Pielou, PD), key species (*Devosia* and *Nocardioides*), and the abundance of aromatic compound degradation functions with each environmental factor based on Mantel’s test. The edge width corresponds to the Mantel’s r statistic for distance correlation, and the edge color indicates statistical significance based on 1,000 permutations.

The primary factors influencing microbial variations were elucidated through the Mantel test (Figure 5). The results showed that soil *p*-CA was negatively correlated with the SOM and AP and positively correlated with the TP and TK. In addition, the soil microbial diversity was predominantly driven by TK, hub genera were influenced by SOM, and ACD was primarily driven by TP and SOM. SOM exerted the greatest impacts on hub genera and ACD.

### 3.6 Structural equation modeling

**Figure 6:**
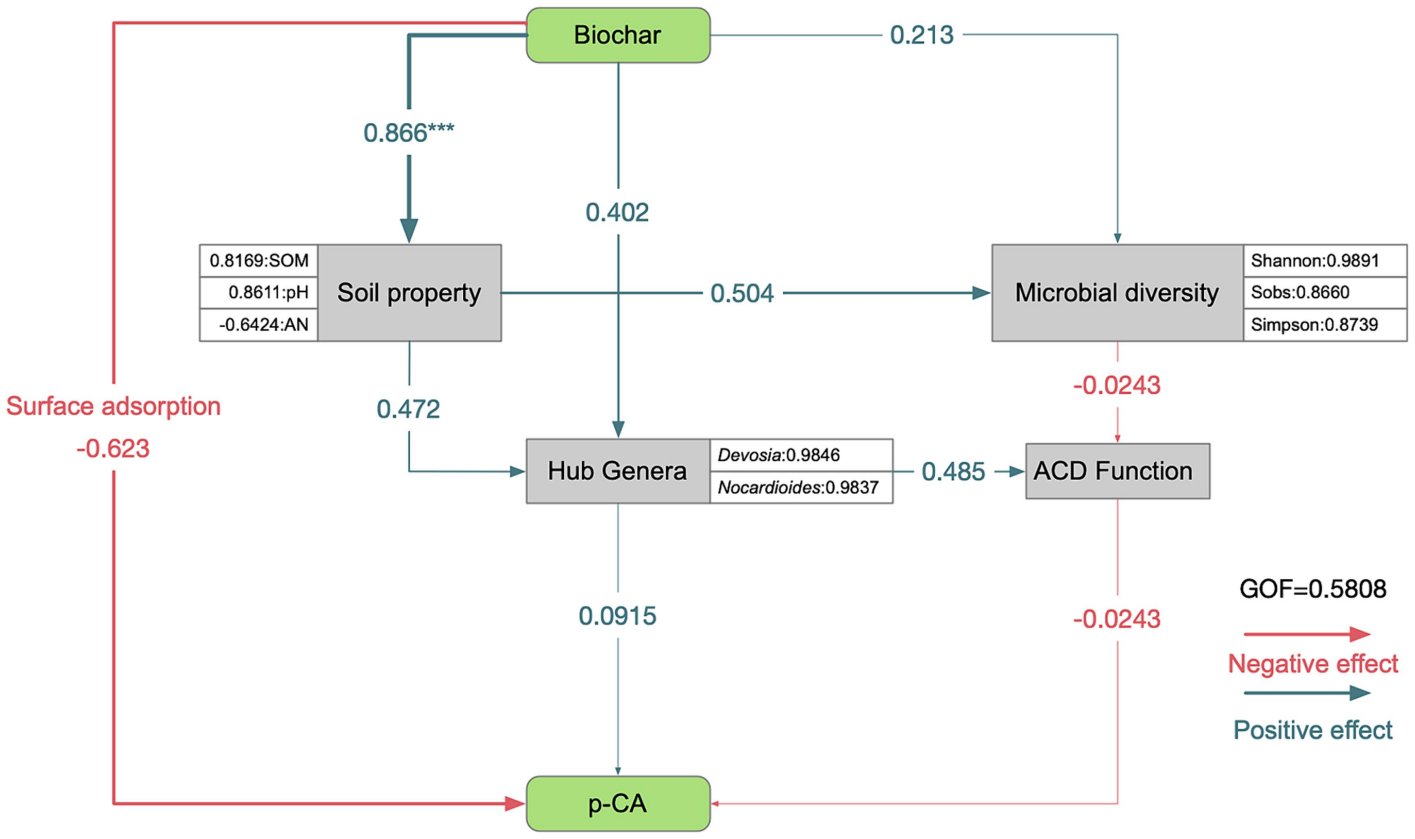
Structural equation modeling (SEM). Path coefficients were estimated using 1,000 bootstraps. Positive and negative effects are indicated by green and orange arrows, respectively; *p < 0.05, **p < 0.01, and ***p < 0.001. The model was evaluated using the goodness of fit (GOF) value. Biochar application was quantified using a binary coding method, with “0” indicating no application and “1” indicating application. Soil properties include TN, TP, TK, SOM, AP, AK, pH, and AN. Microbial diversity metrics include sobs, Shannon, Simpson, Chao, ACE, goods_coverage, Pielou, and PD. Hub genera refer to the absolute abundance of *Devosia* and *Nocardioides*. ACD function represents the relative abundance of aromatic compound degradation in taxon 1. *p*-CA indicates the residual *p*-CA content after 30 days of soil incubation.

A partial least squares path model was employed to analyze the relationships between soil environmental factors, microorganisms, and *p*-CA. The goodness of fit (0.5808) indicated that the model was reliable (Figure 6). The analysis revealed two paths through which BC could reduce *p*-CA. First, BC could directly adsorb *p*-CA (path coefficient = −0.623). Alternatively, BC may increase the SOM content (path coefficient = 0.866, *p* < 0.001), thus enhancing the microbial diversity (path coefficient = 0.504) and the relative abundances of hub genera (path coefficient = 0.472). Both microbial diversity and hub genera affected the ACD function of keystone taxa (path coefficient = −0.0243 and 0.485, respectively), thereby reducing *p*-CA (path coefficient = −0.0243).

## 4 Discussion

### 4.1 BC reduced *p*-CA through chemical adsorption

BC, with its abundant surface functional groups and unique porous structure, is capable of adsorbing harmful substances (Yu et al., 2021; Gong et al., 2022). The primary modes of adsorption, including chemical and physical, largely depend on the characteristics of both BC and the target substance. Laboratory adsorption tests demonstrated that BC effectively adsorbed *p*-CA, with the adsorption capacity increasing over time and reaching equilibrium at 105 min. Additionally, we employed a pseudo-second-order kinetic equation (PKE), the Elovich model (EM), and the intra-particle diffusion model (IDM) to describe the kinetic adsorption process of BC. The results indicated that the PKE model (*R*^2^ > 0.99) most accurately described the adsorption process. Based on the implications of the PKE equation, we hypothesized that the adsorption between BC and *p*-CA likely occurs through covalent bonding facilitated by the sharing or exchange of electrons (Ho and McKay, 2000). While studies specifically on the adsorption mechanism of BC for *p*-CA are not yet available, similar evidence can be drawn from research on BC’s adsorption of cinnamic acid, another phenolic acid. BC may chemically adsorb *p*-CA in two ways: first, through the formation of stable hydrogen bonds between the OH^−^ groups on the BC’s surface and the R-COO^−^ ions from hydrolyzed *p*-CA; and second, by directly binding the oxygen-containing groups on the BC’s surface with undissociated *p*-CA molecules to form a stable structure (Ni et al., 2011). The isothermal adsorption results showed that the maximum adsorption capacity of BC for *p*-CA was 5.205 mg/g. The adsorption was linear at low concentrations but slowed at higher concentrations, consistent with the characteristics of chemical adsorption. This phenomenon may be related to the number of functional groups on the surface of the BC. Each functional group can adsorb only one molecule or atom, and when these groups are saturated, adsorption reaches a monolayer saturation state (Fan et al., 2020). Under solution culture conditions, we confirmed that BC directly reduced the *p*-CA concentration through chemical adsorption, providing a theoretical basis for the potential of BC to mitigate *p*-CA accumulation in soil.

### 4.2 BC indirectly reduced *p*-CA levels by altering the diversity of the microbial community

Soil incubation experiments showed that the application of BC significantly reduced *p*-CA levels in the soil. Numerous studies have indicated that, in addition to directly adsorbing organic pollutants (Hu et al., 2020; Liang et al., 2021), BC can enhance the degradation of pollutants by altering the structure and function of microbial communities (Sigmund et al., 2018; Xiang et al., 2022). In this study, the application of BC notably altered the composition of soil microbial communities and increased the microbial α-diversity. Among these microbial genera, we identified several with potential for degrading phenolic compounds, such as *Streptomyces* (Wu et al., 2019), *Nocardioides* (Maltseva and Oriel, 1997), *Massilia* (Schnell et al., 1991), and *Amycolatopsis* spp. (Grund et al., 1990). This suggests that BC can indirectly reduce soil *p*-CA levels by regulating the soil microbial community, particularly by increasing the abundance and diversity of microbes capable of degrading *p*-CA. These microbial genera could also serve as potential resources for future efforts to enhance biodegradation by directly combining BC with functional microbes. It is well-known that the physicochemical properties of soil are key drivers of changes in the microbial community structure (Xue et al., 2018; Qiang et al., 2021; Sui et al., 2021). In this study, soil pH and the organic matter (OM) content increased significantly, suggesting their important role in driving changes in the microbial community structure. Soil pH and organic matter are considered important indicators of soil microbial activity and quality, and they can significantly influence community structure and function (Aciego Pietri and Brookes, 2009; Zhou et al., 2017; Shao et al., 2019). Previous research has shown that higher soil pH levels favor the growth of genera such as *Streptomyces* (Kontro et al., 2005) and *Massilia* (Raths et al., 2020). An increased organic matter content provides more carbon sources for microbial growth, thereby promoting greater microbial diversity (Tian et al., 2018). Based on the above analysis, we hypothesized that BC application would significantly increase the soil pH and organic matter content, leading to changes in the microbial community and increased diversity that in turn would indirectly enhance the degradation of *p*-CA.

### 4.3 Keystone microbial taxon played a crucial role in the degradation of *p*-CA

An analysis of the microbial taxa offers a better understanding of the role of BC in soil ecological functions (Chen et al., 2019). Using the WGCNA network analysis, we constructed a co-expression network of microbial communities and divided them into five taxa (Figure 4A-a). Among these, taxon 1 was closely associated with *p*-CA levels (Figure 4A-b, *r* = −0.520, *p* = 0.027), suggesting that this taxon might be a keystone taxon for *p*-CA degradation. The species composition analysis supported this view, revealing that taxon 1 had higher abundances of Patescibacteria (Sun et al., 2023) and Chloroflexi (Colatriano et al., 2018) species, both of which have organic pollutant degradation capabilities, compared to other taxa. Additionally, the application of BC increased the proportion of the soil ACD function, likely due to the alterations BC made to the soil environment. To adapt to environmental changes, keystone taxa selectively adjust the relative abundances of their members, promoting or inhibiting certain functions within the group (Van Der Heijden et al., 1998; van der Heijden et al., 2015), and secreting metabolites, antibiotics, or toxins to shape a favorable microbial community (Hajishengallis et al., 2012; Shetty et al., 2017). Therefore, we speculated that taxon 1 has a *p*-CA degradation function, and that the application of BC enhances this function. By establishing correlations among the taxon, species, and *p*-CA, we identified *Devosia* and *Nocardioides* within taxon 1 as key genera. There is also evidence that these two genera can reduce the organic content by secreting degrading enzymes (Saito et al., 1999; Benedek et al., 2022; Xiong et al., 2023; Li et al., 2024), potentially playing key roles in soil C/N cycling and the remediation of organically polluted soils (Tao et al., 2022; Siebielec et al., 2023). Thus, we have reason to believe that the enhancement of *p*-CA degradation by BC application is closely related to *Devosia* and *Nocardioides*.

In summary, BC-induced changes in the soil environment prompted the keystone taxon to adjust its composition to enhance overall degradation functions. Currently, there is limited research on how BC affects the ACD function abundance. We speculated that BC may influence key functional regulation by altering the abundance of specific members within the taxon (e.g., *Devosia* and *Nocardioides*), ultimately reducing *p*-CA levels. Unfortunately, our hypothesis was primarily based on statistical results of the OTU relative abundance that only indicated potential interactions and may not have fully reflected actual microbial interactions. However, our findings further suggest the significant potential of keystone taxa in assessing soil microecological responses to environmental changes, offering new perspectives for exploring the relationship between soil environments and microorganisms.

## 5 Conclusion

In the present study, BC demonstrated the ability to chemisorb *p*-CA, with a maximum adsorption capacity of 5.205 mg/g. BC significantly increased the pH, SOM content, and bacterial community diversity in soil. Notably, BC application influenced the abundance of microbial taxa, with taxon 1 emerging as the keystone taxa closely associated with the reduction of *p*-CA. BC may regulate microbial composition and diversity by modifying the SOM, thus altering the relative abundances of specific members within the hub genera (*Devosia* and *Nocardioides*) and enhancing the ACD function. This ultimately leads to the degradation of *p*-CA. Taken together, BC demonstrated the ability to directly adsorb *p*-CA through chemisorption and indirectly promote *p*-CA degradation via regulating the functions of keystone taxa. These findings provide a novel perspective to enhance the knowledge of the mechanisms underlying the BC-mediated alleviation of accumulated toxic substances.

## Data Availability

The datasets presented in this study can be found in online repositories. The names of the repository/repositories and accession number(s) can be found at: https://ngdc.cncb.ac.cn/gsa, CRA013351.

## Footnotes

^1^ https://ngdc.cncb.ac.cn/gsa
