# Supplementary material for: Biochar regulates the functions of keystone taxa to reduce p-coumaric acid accumulation in soil
Source: Front Microbiol. 2024 Sep 11;15:1458185. doi: 10.3389/fmicb.2024.1458185 (PMC11425655; doi:10.3389/fmicb.2024.1458185)
Supplement: Supplementary file 1 [file Data_Sheet_1.pdf]

Supplementary Material

Biochar regulates the functions of keystone taxa to reduce *p*-coumaric acid accumulation in soil

1 Supplementary Figures

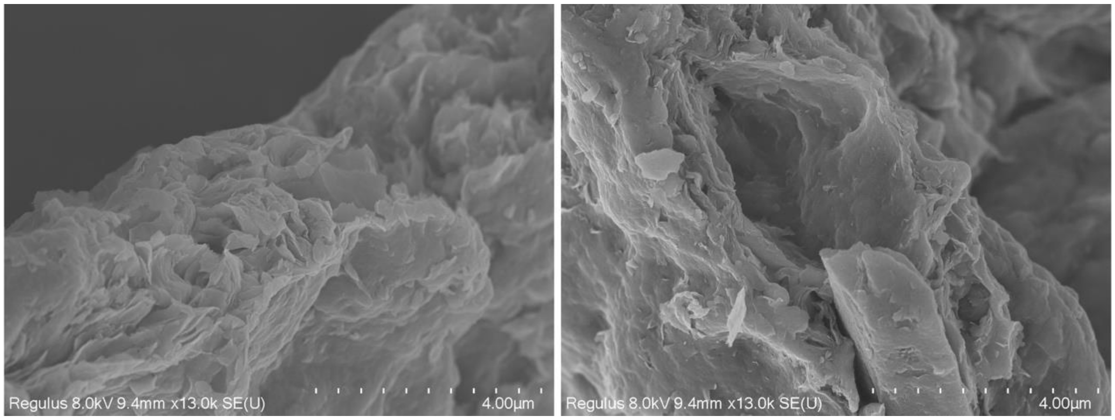

Figure S1 Biochar microstructure under scanning electron microscope.

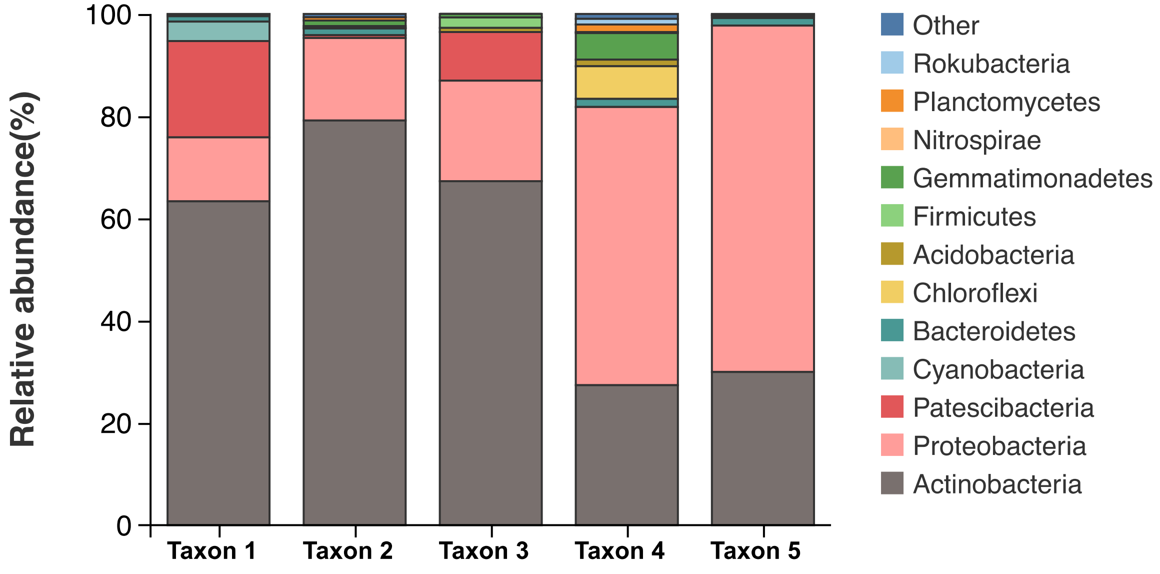

Figure S2 Relative abundances of phyla in taxa.

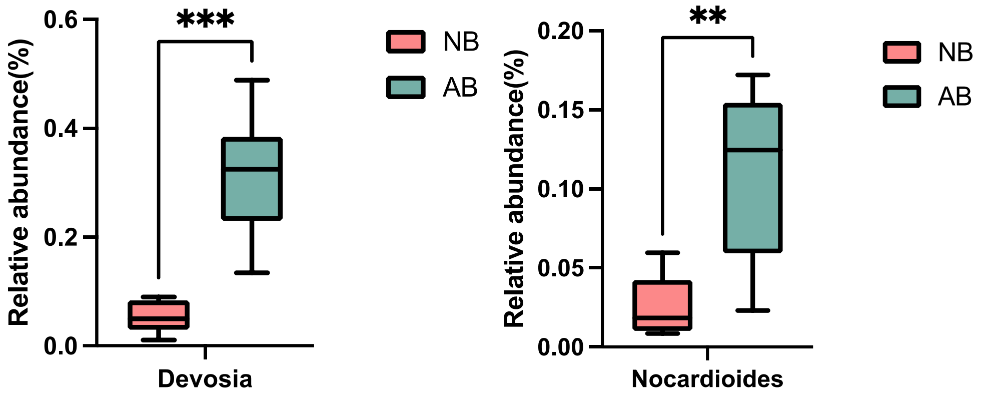

Figure S3 Relative abundances of *Devosia* and *Nocardioides* in NB and AB. NB, treatment without biochar; AB, treatment with biochar.

2 Supplementary Tables

Table S1 Physiochemical properties of soil and biochar

	pH	AN (mg/kg)	AP (mg/kg)	AK (mg/kg)	SOM (g/kg)	SOC (g/kg)	TK (g/kg)	TP (g/kg)	TN (g/kg)
Soil	5.73	69.76	27.78	112.94	12.08	—	1.48	1.58	0.31
BC	10.18	71.50	10.52	711.75	—	313.81	11.33	0.39	2.08

Note: AN, soil alkali-soluble nitrogen; AP, soil available phosphorus; AK, soil available potassium; SOM, soil organic matter; SOC, biochar organic carbon; TK, soil total potassium; TP, soil total phosphorus; TN, soil total nitrogen; BC, biochar.

Table S2 Parameters of artificial climate chamber

Time	Temperature (°C)	Relative humidity (RH,%)	Illumination (Lx)
08:00–09:00	22	70	2000
09:00–10:00	24	70	3500
10:00–11:00	26	70	5000
11:00–12:00	28	70	6500
12:00–13:00	28	70	8000
13:00–14:00	30	70	9000
14:00–15:00	30	70	9000
15:00–16:00	30	70	8000
16:00–17:00	28	70	6500
17:00–18:00	26	70	5000
18:00–19:00	26	70	3500
19:00–20:00	26	80	2000
20:00–21:00	24	80	0
21:00–22:00	24	80	0
22:00–23:00	22	80	0
23:00–24:00	22	80	0
00:00–01:00	20	80	0
01:00–02:00	20	80	0
02:00–03:00	20	80	0
03:00–04:00	20	80	0
04:00–05:00	20	80	0
05:00–06:00	20	80	0
06:00–07:00	20	80	0
07:00–08:00	20	80	0

Table S3 Mobile phases of high-performance liquid chromatography

Time (min)	Mobile phase A (%)	Mobile phase B (%)	Flow rate (mL·min^{-1})	Column oven temperature (°C)	Detection wavelength (nm)
0	30	70			
6	30	70			
10	50	50			
15	70	30	1	30	320
17	50	50			
18	30	70			
20	30	70			

Table S4 Model fitting parameters

Module	Parameters	Value
Langmuir	Qmax (mg·g^{-1})	5.205
	K_L	0.117
	R^2	0.932
Freundlich	K_F	0.6
	1/n	0.646
	R^2	0.891
PKE	K (g·mg^{-1}·min^{-1})	0.051
	Qe (mg·g^{-1})	1.394
	R^2	0.996
EM	a	0.11
	b	0.242
	R^2	0.99
IDM	Kp (g·mg^{-1}·min$^{-1/2}$)	0.065
	c	0.568
	R^2	0.935

Note: PKE is the pseudo-second-order kinetic model; EM is the Elovich model, IDM is the intra-particle diffusion model; K is the rate constant of pseudo-second-order kinetic model; Qe is the equilibrium adsorption capacity; k_p is the rate constant of intra-particle diffusion model; a, b, and c are constants; Qmax is the maximum adsorption capacity; K_L is the Langmuir constant, with K_L = 0 indicating irreversible adsorption, $0<K_L<1$ favorable adsorption, K_L = 1 linear adsorption, and $K_L>1$ unfavorable adsorption; and K_F and n are the Freundlich constants, with high K_F indicating high adsorption capacity of biochar.

Table S5 Effects of biochar application on soil physicochemical properties

Treatment	TN (g/kg)	TP (g/kg)	TK (g/kg)	AP (mg/kg)	AK (g/kg)
NB	0.45±0.11a	2.00±0.19a	1.72±0.34a	27.16±1.65a	1.35±0.11a
AB	0.44±0.09a	1.93±0.18a	1.65±0.49a	29.13±3.14a	1.27±0.21a

Note: TN, soil total nitrogen; TP, soil total phosphorus; TK, soil total potassium; AP, soil available phosphorus; AK, soil available potassium; NB, no BC treatment is applied; AB, BC treatment is applied.

Table S6 Number of taxa in taxa

taxa	OTU	Phylum	Class	Order	Family	Genus	Species
Taxon 1	107	7	14	23	28	29	3
Taxon 2	87	8	12	24	25	30	5
Taxon 3	31	6	9	17	19	12	1
Taxon 4	123	9	21	34	46	45	6
Taxon 5	43	6	9	11	13	19	3

Note: OTU, operational taxonomic unit.

Table S7 Topological parameters of correlation networks

Treatment	Node	Edge	Average degree	Average weighted degree	Diameter	Density	Modularity	Average clustering coefficient	Average path length
NB	363	1414	7.791	7.071	16	0.022	0.798	0.559	6.9
AB	468	1940	8.291	7.392	23	0.018	0.692	0.490	7.052

Note: NB, no BC treatment; AB, with BC treatment.
